# Chemical Exposures: Cancer and TCDD: The Mitochondrial Connection

**DOI:** 10.1289/ehp.116-a112

**Published:** 2008-03

**Authors:** M. Nathaniel Mead

During the Vietnam War, from 1961 to 1971, U.S. military forces sprayed millions of gallons of the herbicide Agent Orange over vast tracts of Southeast Asian jungle, mainly in an effort to remove foliage and expose enemy troops. Troops were exposed to TCDD that contaminated the Agent Orange, and since the 1970s, elevated blood TCDD concentrations have been implicated in many cancers, skin rashes, and other health problems experienced by Vietnam veterans. Although TCDD is carcinogenic, it is not directly genotoxic. A report in the 8 January 2008 *Proceedings of the National Academy of Sciences* now demonstrates one of the ways that TCDD may promote cancer’s growth and spread.

The new study describes a novel mechanism of TCDD action that focuses on the mitochondria: “We found that TCDD induces tumor cell proliferation and invasion by directly acting on mitochondrial transcription machinery and inducing mitochondrial respiratory stress,” says principal investigator Narayan G. Avadhani, a biochemistry professor at the University of Pennsylvania. Such mitochondrial dysfunction inhibits apoptosis in malignant cells and increases the invasive potential of cancer. Mitochondrial dysfunction is also associated with conditions such as heart disease, diabetes, obesity, blindness, deafness, kidney disease, and neurodegenerative disorders, as well as with aging.

“[The respiratory stress-signaling] cascade culminates in the activation of a large number of nuclear genes that affect various cellular processes including cell metabolism, proliferation, and apoptosis,” says lead author Gopa Biswas, a researcher in Avadhani’s lab. “We have now established that TCDD alters cellular morphology and physiology through a similar mechanism.”

It is generally accepted that adverse effects of TCDD result from its activation of the Ah receptor, with effects occurring at very low exposures. In the presence of TCDD, the Ah receptor has been shown to either induce or suppress the transcription of numerous genes that have been linked with cancer development via changes in tumor suppressor proteins, oncogenes, growth factors, and cell cycle proteins, among other factors.

Mitochondrial dysfunction may entail a more fundamental mechanism. It appears that TCDD-induced mitochondrial stress signaling in cancer cells is propagated in part through the Ah receptor but also acts through mechanisms that are independent of the Ah receptor, such as by inducing protein kinase C and extracellular signal–regulated kinases.

“Our findings show that at subtoxic levels of ten to fifty nanomolar, TCDD is sufficient to cause mitochondrial dysfunction and induce the signaling cascade,” says Avadhani. “These results raise concerns over the adverse health implications of dioxins and PCBs even at very low levels.”

In both animal and human studies (notably epidemiologic analyses of cancer rates following the 1976 industrial accident in Seveso, Italy), TCDD exposure has increased cancer incidence and mortality at all cancer sites rather than at a few specific sites. In 1997, the International Agency for Research on Cancer upgraded TCDD to a Group 1 human carcinogen on the basis of mechanistic data. Considering subsequent dose–response assessments for TCDD and cancer, Kyle Steenland, a professor of environmental and occupational health at Emory University, and colleagues argued in the September 2004 issue of *EHP* that “TCDD exposure levels close to those in the general population may be carcinogenic and argue for caution in setting the upper ranges of long-term permissible exposure to dioxins.”

The present study is limited in that it involved skeletal myoblasts, not living organisms. “These findings are significant but unfortunately provide no *in vivo* data showing tumor progression in animals due to loss of mitochondrial function by TCDD,” says Keshav K. Singh, a cancer geneticist at Roswell Park Cancer Institute in Buffalo, New York. “At a minimum, xenograft studies in mice are needed.” Avadhani now plans to study the precise mitochondrial targets of different polychlorinated biphenyls (a related group of compounds) that lead to reduced mitochondrial transcription and then examine the implications of this pathway in tumor progression *in vivo*. He sees possible implications for the prevention of breast, pancreatic, and other endocrine cancers.

Recognition that the carcinogenic effects of environmental toxicants may originate in disruption of mitochondrial biology could prove important for the future development of cancer prevention and treatment procedures related to TCDD and other dioxin exposures. “The new findings suggest that the risk of cancer may be reduced by avoiding or lowering exposure to environmental mitochondrial toxicants as well as [possibly] by optimizing mitochondrial energy metabolism by nutritional and medicinal means,” says Egil Fosslien, a pathology professor emeritus at the University of Illinois at Chicago.

## Figures and Tables

**Figure:**
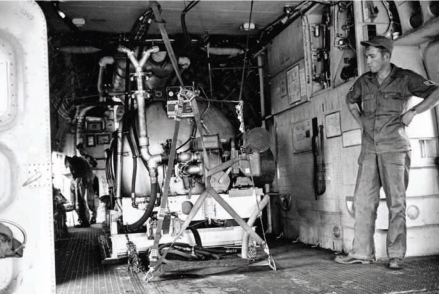
A U.S. Air Force crew member stands by a defoliant mixing machine inside one of three C-123 aircraft used to clear jungle growth in South Vietnam; August 1963.

